# Supplementary material for: Turn-On Fluorescence Aptasensor on Magnetic Nanobeads for Aflatoxin M1 Detection Based on an Exonuclease III-Assisted Signal Amplification Strategy
Source: Nanomaterials (Basel). 2019 Jan 16;9(1):104. doi: 10.3390/nano9010104 (PMC6359137; doi:10.3390/nano9010104)
Supplement: Supplementary file 1 [file nanomaterials-09-00104-s001.pdf]

Turn-On Fluorescence Aptasensor on Magnetic Nanobeads for Aflatoxin M1 Detection Based on an Exonuclease III-Assisted Signal Amplification Strategy

Fuyuan Zhang [1,2,3,†] Linyang Liu [2,†], Shengnan Ni[4], Jiankang Deng [3], Guo-Jun Liu [5,6], Ryan Middleton [5,6], David W. Inglis [1], Shuo Wang [3,7] and Guozhen Liu [1,2,4,*]

1 ARC Centre of Excellence in Nanoscale Biophotonics (CNBP), Macquarie University, North Ryde 2109, NSW, Australia; fuyuan.zhang@aliyun.com (F.Z.); david.inglis@mq.edu.au (D.W.I.)
2 Graduate School of Biomedical Engineering, Faculty of Engineering, University of New South Wales, Sydney 2052, NSW, Australia; linyang.liu@unsw.edu.au
3 State Key Laboratory of Food Nutrition and Safety, Key Laboratory of Food Nutrition and Safety, Ministry of Education of China, Tianjin University of Science and Technology, Tianjin 300457, China; dengjk1989@163.com (J.D.); s.wang@tust.edu.cn (S.W.)
4 International Joint Research Center for Intelligent Biosensor Technology and Health, College of Chemistry, Central China Normal University, Wuhan 430079, China; 15527604959@163.com
5 Australian Nuclear Science and Technology Organization, Lucas Heights, NSW 2234, Australia; gdl@ansto.gov.au (G.-J.L.); rym@ansto.gov.au (R.M.)
6 Discipline of Medical Imaging & Radiation Sciences, Faculty of Medicine and Health, Brain and Mind Centre, University of Sydney, 94 Mallett Street, Camperdown, NSW 2050, Australia
7 Tianjin Key Laboratory of Food Science and Health, School of Medicine, Nankai University, Tianjin 300071, China
* Correspondence: guozhen.liu@unsw.edu.au
† These authors contributed equally to this work.

Conjugation of Magnetic beads to the Biotinylated Aptamers.

To test the capability of the magnetic beads, an excess of aptamers (50 μL, 5 μM) was added to 50 μL 1 mg mL^{-1} of streptavidin-coated magnetic beads. The conjugation reaction was carried out under gentle mixing for 30 min. The captured aptamers were separated together with the magnetic beads in a magnetic field. The concentration of free aptamers left in the solution were measured with the NanoDrop, which showed a concentration of 1.6 μM in the solution. It indicated that 0.9 μM of aptamers were captured by 1 mg/mL of magnetic beads. Subsequently, the resultant magnetic beads were added to 30 μL 4.7 μM of C-strand DNA solution. After a gentle mixing for 30 min, 2.3 μM of C-strand were detected in the left solution, which showed 2.4 μM of aptamer/C duplex were formed onto 1 mg/mL of magnetic nanobeads.

Table S1. The DNA sequences used in the experiments.

DNAs	Sequences
Aptamer	5'-ACTGCTAGAGATTTTCCACAT-C6-biotin-3'
C-strand	5'-CAAACTCTCTATCAGTGG-3'
T-strand	5'-AAAACCCAAAACCCAAAACCCACTGATAGAGAGTTTG-3'
G-strand	5'-CTAGCAGAGGGTTTTGGGTTTTGGGTTTTGGGAGCTA-3'

Table S2. The comparisons of the previous fluorescence aptasensors and our aptasensor.

Developed aptasensors	Strategy	Detection range	LOD
Guo et al[1]	A target-induced DNA machine amplification fluorescence assay	0.06-0.6 nM	0.01 ng mL^{-1}
Yin et al[2]	A G-quadruplex-specific fluorescence probe amplification method	0.01-2.0 ng mL^{-1}	17.79 ng kg^{-1}
Seyyed et al[3]	An electrochemiluminescence (ECL) aptasensor	10-200 ng mL^{-1}	0.05 ng mL^{-1}
Atul et al[4]	A structure-switching signaling aptamer assay based on the FAM-TAMRA quenching-dequenching mechanism	1-2000 ng kg^{-1}	5.0 ng kg^{-1}
Our method	A fluorescence aptasensor based on a magnetic nanomachine and an Exonuclease III-assisted signal amplification strategy	0.01-2 ng mL^{-1}	9.73 ng kg^{-1}

Reference

1. Guo, T.; Lin, X.; Liu, Y.; Deng, J.; Qian, P.; Lyu, Y.; Zhang, Z.; Wang, S., Target-induced DNA machine amplification strategy for high sensitive and selective detection of biotoxin. *Sensors and Actuators B: Chemical* **2018,** *262*, 619–624.
2. Yin, J.; Liu, Y.; Wang, S.; Deng, J.; Lin, X.; Gao, J., Engineering a universal and label-free evaluation method for mycotoxins detection based on strand displacement amplification and G-quadruplex signal amplification. *Sensors and Actuators B: Chemical* **2018,** *256*, 573–579.
3. Khoshfetrat, S. M.; Bagheri, H.; Mehrgardi, M. A., Visual electrochemiluminescence biosensing of aflatoxin M1 based on luminol-functionalized, silver nanoparticle-decorated graphene oxide. *Biosens Bioelectron* **2018,** *100*, 382–388.
4. Sharma, A.; Catanante, G.; Hayat, A.; Istamboulie, G.; Ben Rejeb, I.; Bhand, S.; Marty, J. L., Development of structure switching aptamer assay for detection of aflatoxin M1 in milk sample. *Talanta* **2016,** *158*, 35–41.
